# Preoperative Breast Magnetic Resonance Imaging: An Ontario Health (Cancer Care Ontario) Clinical Practice Guideline

**DOI:** 10.3390/curroncol30070463

**Published:** 2023-06-30

**Authors:** Derek Muradali, Glenn G. Fletcher, Erin Cordeiro, Samantha Fienberg, Ralph George, Supriya Kulkarni, Jean M. Seely, Rola Shaheen, Andrea Eisen

**Affiliations:** 1 Department of Medical Imaging, University of Toronto, Toronto, ON M5T 1W7, Canada; supriya.kulkarni@uhn.ca; 2 Program in Evidence-Based Care, Department of Oncology, McMaster University, Hamilton, ON L8S 4L8, Canada; gfletche@mcmaster.ca; 3 Department of Surgery, University of Ottawa, Ottawa, ON K1N 6N5, Canada; ecordeiro@toh.ca; 4 Ontario Health (Cancer Care Ontario), Toronto, ON M5G 2L3, Canada; samantha.fienberg@ontariohealth.ca; 5 Department of Surgery, University of Toronto, Toronto, ON M5T 1P5, Canada; ralph.george@unityhealth.to; 6 Department of Radiology, The Ottawa Hospital, University of Ottawa, Ottawa, ON K1N 6N5, Canada; jeseely@toh.ca; 7 Department of Radiology, Queen’s University, Kingston, ON K7L 3N6, Canada; rshaheen@prhc.on.ca; 8 Diagnostic Imaging, Peterborough Regional Health Centre, Peterborough, ON K9J 7C6, Canada; 9 Department of Medical Oncology, Odette Cancer Centre, Sunnybrook Health Sciences Centre, Toronto, ON M4N 3M5, Canada

**Keywords:** breast cancer, magnetic resonance imaging, practice guideline

## Abstract

Background: The use of preoperative breast magnetic resonance imaging (MRI) after the diagnosis of breast cancer by mammography and/or ultrasound is inconsistent. Methods: After conducting a systematic review and meta-analysis comparing preoperative breast MRI versus no MRI, we reconvened to prepare a clinical practice guideline on this topic. Results: Based on the evidence that MRI improved recurrence, decreased the rates of reoperations (re-excisions or conversion mastectomy), and increased detection of synchronous contralateral breast cancer, we recommend that preoperative breast MRI should be considered on a case-by-case basis in patients diagnosed with breast cancer for whom additional information about disease extent could influence treatment. Based on stronger evidence, preoperative breast MRI is recommended in patients diagnosed with invasive lobular carcinoma for whom additional information about disease extent could influence treatment. For both recommendations, the decision to proceed with MRI would be conditional on shared decision-making between care providers and the patient, taking into account the benefits and risks of MRI as well as patient preferences. Based on the opinion of the Working Group, preoperative breast MRI is also recommended in the following more specific situations: (a) to aid in surgical planning of breast conserving surgery in patients with suspected or known multicentric or multifocal disease; (b) to identify additional lesions in patients with dense breasts; (c) to determine the presence of pectoralis major muscle/chest wall invasion in patients with posteriorly located tumours or when invasion of the pectoralis major muscle or chest wall is suspected; (d) to aid in surgical planning for skin/nipple-sparing mastectomies, autologous reconstruction, oncoplastic surgery, and breast conserving surgery with suspected nipple/areolar involvement; and (e) in patients with familial/hereditary breast cancer but who have not had recent breast MRI as part of screening or diagnosis.

## 1. Introduction

Suspected breast cancer based on clinical examination or screening mammography is generally confirmed by diagnostic mammography (with or without ultrasound) and biopsy. Surgery may be preceded by further advanced imaging of higher sensitivity or diagnostic utility, with contrast-enhanced breast magnetic resonance imaging (CE-MRI, often referred to as MRI) being the most widely used to characterize the locoregional extent of breast cancer.

Breast MRI has a sensitivity for detecting cancer of greater than 90% and as high as 97% to 100% [1,2,3] in some studies of screening or for preoperative use after diagnosis. Studies published prior to 2000 had suggested poor sensitivity for ductal carcinoma in situ (DCIS); however, with improved equipment and radiologist expertise, this is no longer the case [4,5,6]. MRI specificity depends on study populations, technical methods, and criteria for interpretation. It is generally greater than 70%, and up to 97% has been reported [1]. The American College of Radiology Breast Imaging Reporting and Data System (BI-RADS) Atlas provides standardized terminology and reporting to assist in interpretation and sets a benchmark for specificity in screening MRI at 85% to 90% [7].

The use of MRI in screening and surveillance is considered standard of care for individuals at higher risk of breast cancer due to genetic factors or previous radiation exposure for another cancer [8,9]. Some recent guidelines include personal history or dense breasts as high-risk factors warranting consideration of an MRI [10,11]. Cancer screening is dealt with in several other guidelines and was not included in the current work.

It has been established that MRI has higher sensitivity than mammography and ultrasound, as illustrated by its incorporation into high-risk screening; however, there is less consensus on whether the additional information provided by preoperative MRI subsequent to the cancer diagnosis improves patient outcomes. Use of breast MRI beyond screening is the topic of guidelines by the European Society of Breast Cancer Specialists (EUSOMA) [12], the European Society of Breast Imaging (EUSOBI) [13], and the Institut national d’excellence en santé et en services sociaux (INESSS; Quebec, Canada) [14], and a practice parameter by the American College of Radiology (ACR) [15]. Also relevant are the Canadian Association of Radiologists imaging guideline [16], which has a section on MRI, and the evidence review/medical policies by Blue Cross/Blue Shield [17]. General breast cancer guidelines such as those by the National Comprehensive Cancer Network (NCCN) [18] also have recommendations on MRI use; several of these have only a few points regarding MRI and may not be based on a review of the primary literature. It was determined that these guidelines either did not cover the most recent studies, had a different focus, or did not conduct a comprehensive review. We therefore conducted a systematic review and meta-analysis [19,20,21] comparing outcomes such as re-operation rates, recurrence, and survival with versus without preoperative breast MRI, followed by the development of recommendations as reported in this clinical practice guideline.

## 2. Materials and Methods

### 2.1. Background

The Program in Evidence-Based Care (PEBC) is an initiative of the Ontario provincial cancer system, Ontario Health (Cancer Care Ontario), supported by the Ontario Ministry of Health. The PEBC produces evidence-based and evidence-informed guidance documents using the methods of the Practice Guidelines Development Cycle [22,23]. This process includes a systematic review, interpretation of the evidence, and draft recommendations by the Working Group; internal review by content and methodology experts; and external review by Ontario clinicians and other stakeholders. PEBC guideline recommendations are based on evidence of the desirable and undesirable effects of an intervention or the accuracy of a test and take into account the certainty of the evidence. PEBC guideline development methods are described in more detail in the *PEBC Handbook* and the *PEBC Methods Handbook* (https://www.cancercareontario.ca/en/guidelines-advice/types-of-cancer/breast, accessed on 1 May 2023).

### 2.2. Guideline Objective

The primary goal was to make recommendations about whether preoperative breast magnetic resonance imaging (MRI) should be added to conventional imaging (mammography and/or ultrasound) in patients with newly diagnosed breast cancer and to make recommendations about specific indications if evidence allowed.

### 2.3. Research Question

In patients with newly diagnosed breast cancer, does additional information on the extent of disease obtained by preoperative breast MRI after mammography and/or ultrasound (a) change the type or extent of surgery (breast conserving surgery (BCS), unilateral or bilateral mastectomy), the type or extent of radiation therapy, or the use of adjuvant therapy; or (b) improve patient outcomes such as recurrence, disease-free survival or event-free survival, distant metastasis-free survival, overall survival, rates of re-excision or re-operation, or quality of life?

### 2.4. Target Population

The target population is patients already diagnosed with breast cancer of any stage for whom additional information on disease location or extent in the breast obtained prior to surgery may influence staging, treatment, or prognosis. The guideline does not address patients diagnosed with breast cancer but without an identified cancerous lesion in the breast (occult breast cancer) or patients undergoing neoadjuvant therapy prior to surgery. Imaging for distant metastasis is the topic of a separate guideline [24].

### 2.5. Development Process

This guideline is based on a systematic review and meta-analysis originally completed in December 2021 [19]. The systematic review was revised to incorporate study updates until July 2022 and to include additional quality assessment using the Grading of Recommendations, Assessment, Development and Evaluation (GRADE) approach to aid in developing the clinical practice recommendations [20]. An integrated version of the systematic review is also available [21].

The Working Group (the authors of this article) was responsible for reviewing the evidence base, drafting the guideline recommendations, and responding to comments received during the document review process. The Working Group had expertise in radiology, surgery, medical oncology, and health research methodology.

### 2.6. Literature Search

Embase, MEDLINE, the Cochrane Central Register of Controlled Trials, and the Cochrane Database of Systematic Reviews were searched until 3 July 2019 and updated until 18 January 2021. A targeted search was conducted in July 2022 to identify any additional publications related to the included RCTs and studies identified as ongoing. Studies had to be comparative studies of MRI versus no MRI after a diagnosis of breast cancer and report rates of survival, recurrence, re-excision, reoperation, or mastectomy. One author (GGF) reviewed all studies, and co-authors were consulted in cases of uncertainty regarding inclusion.

Included were 8 randomized controlled trials (RCTs), 1 prospective cohort study, and 42 retrospective studies. The patient population was limited to those with an initial treatment plan of breast conserving surgery in 17 trials (6 RCTs). The retrospective studies included 8 with propensity-matched controls, 4 with historical or equivalent controls, 15 with multivariable/multivariate analysis of data from a single or small number of institutions, and 15 using cancer registry data and multivariable/multivariate analysis. A series of forest plots created using RevMan [25] provide graphical summaries to aid in the interpretation of the tabulated results.

Data was extracted from the included studies. Odds ratios (OR) or hazard ratios (HR) were expressed with a ratio of <1.0, indicating that the experimental group (MRI use) had a more favourable outcome than the control group. The exception to this was the case of synchronous contralateral breast cancer (CBC) detection (identified at the same time or sometimes defined as occurring within 6 months of the index cancer). Higher detection is considered a favourable outcome, but the convention is to report increased detection with HR >1.0. The risk of bias for randomized studies was assessed per outcome and per study using the Cochrane risk-of-bias (RoB) tool (revised version RoB2) for RCTs and ROBINS-I for non-RCTs, as outlined in the Cochrane Handbook for Systematic Reviews of Interventions [26]. The GRADE approach was used to facilitate recommendation development. The full GRADE evaluation, including risk of bias assessment, summary of findings tables, GRADE profiles, and standardized statements for each outcome, including levels of certainty, has been reported [20]. Both the original review [19] and update [20] should be consulted for further details; the review portions have been merged in a subsequent publication [21].

### 2.7. Recommendation Development and Review

The Working Group evaluated the systematic review and developed clinical practice recommendations. The document was then reviewed by a Patient- and Caregiver-Specific Consultation Group consisting of five people with personal experience with cancer (patients/survivors/caregivers) who participated as Patient Consultation Group members. The internal review consisted of reviews by an Expert Panel of eight content experts and by the PEBC Report Approval Panel, a three-person panel with methodology expertise. All participants approved of the document; comments were considered by the Working Group in revising the document.

Feedback on the approved draft guideline was obtained from content experts and the target users through two processes. Through the Targeted Peer Review, three individuals with content expertise were identified and asked to review and provide feedback on the guideline document. Through Professional Consultation, relevant care providers and other potential users of the guideline were contacted, and 41 provided feedback on the guideline recommendations through a brief online survey and additional comments. The Working Group considered all feedback in making final revisions.

## 3. Recommendations and Key Evidence

It has been established that breast MRI can provide additional information on lesion presence, size, location, and distribution; it is less certain in what circumstances this will lead to better patient outcomes. There are both potential benefits and harms to consider (see Table 1), and the relative importance will vary depending on patient and disease characteristics; technical considerations related to equipment and radiology team expertise; and system considerations such as cost, availability of equipment and staff, and wait lists for MRI and other procedures and consultations.

### 3.1. Recommendation 1

- Preoperative breast MRI **should be considered** on a case-by-case basis in patients diagnosed with breast cancer for whom additional information about disease extent could influence treatment. The ensuing decision of whether to conduct an MRI should be made in consultation with the patient and must take into account the balance of benefits and risks and patient preferences.
- Stronger recommendations for specific situations are provided in Recommendations 2 and 3.

#### 3.1.1. Qualifying Statements for Recommendation 1

- Benefits and harms (see Key Evidence and Table 1) may vary depending on patient and disease characteristics such as breast density, tumour size, tumour stage, number and distribution of tumours (multicentric or multifocal), subtype of cancer, type of surgery being considered or preferred, adjuvant treatment, and patient factors/comorbidities.
- System issues such as MRI availability may result in treatment delays that may modify the decision.
- “Treatment” in the recommendation includes surgery as well as radiation and systemic treatment.
- In patients with a strong preference for mastectomy or with contraindications to BCS, MRI is unlikely to change surgical planning in the ipsilateral breast. Breast MRI may still impact treatment if mammographically occult CBC is detected.
- Contrast-enhanced mammography (contrast-enhanced spectral mammography, contrast-enhanced digital mammography), diffusion-weighted imaging (DWI) MRI, magnetic resonance spectroscopy, or other advanced imaging techniques are known to provide additional information beyond that of conventional imaging and may be suitable instead of or in addition to CE-MRI. Potential adverse effects due to contrast agent and radiation exposure vary among these techniques, whereas many other potential benefits and harms in Table 1 would be relevant. These are mentioned briefly in the systematic review, but the evaluation was outside of scope. They are less widely available, and there is much less evidence regarding their effect on patient outcomes.

#### 3.1.2. Key Evidence for Recommendation 1

The literature review compared patients with and without preoperative MRI and reported the following results:

##### Recurrence

- Use of MRI is associated with a reduction of recurrence of any type ( HR = 0.77, 95% confidence interval [CI] = 0.65 to 0.90) [moderate level of certainty]. Approximate recurrence: 8.2% versus 10.5%; 2.3% less (1% to 3.6% fewer).

##### Contralateral Cancer

- Use of MRI is associated with an increase in detection of synchronous CBC (prior to initial surgery) (HR = 2.52, 95% CI = 1.75 to 3.62; HR > 1 indicates increased detection with MRI) [moderate level of certainty]. Approximate synchronous CBC detection: 4.7% versus 1.9%; 2.8% more (1.4% to 4.8% more).
- Use of MRI is associated with a slight reduction in metachronous CBC (HR = 0.71, 95% CI = 0.59 to 0.85) [moderate level of certainty]. Approximate metachronous CBC: 1.7% versus 2.4%; 0.7% fewer (0.4% to 1.0% fewer).

##### Conversion Mastectomy

- Use of MRI is associated with a reduction in the rate of conversion mastectomy OR = 0.76, 95% CI = 0.58 to 0.99) [low level of certainty]. Approximate conversion mastectomy rate: 5.5% versus 7.1%; 1.6% fewer (95% CI = 0.1% to 2.9% fewer).

##### Positive Margins

- Use of MRI reduced the rate of positive margins in studies with low or low-moderate risk of bias (OR = 0.57, 95% CI = 0.36 to 0.89) [moderate level of certainty]. Approximate rate of positive margins: 6.5% versus 10.9%; 4.4% fewer (95% CI = 1.1% to 6.7% fewer).

##### Reoperations and Re-Excisions

- Use of MRI is associated with a reduction in the rate of reoperation (OR = 0.73, 95% CI = 0.63 to 0.85) [low level of certainty]. Approximate rate of reoperation: 14.4% versus 18.7%; 4.3% fewer (95% CI = 2.3% to 6.0% fewer).
- Use of MRI is associated with a reduction in the rate of re-excision (OR = 0.63, 95% CI = 0.45 to 0.89) [low level of certainty]. Approximate rate of re-excision: 6.9% versus 10.5%; 3.6% fewer (95% CI = 1.0% to 5.5% fewer).

##### Mastectomy Rates

- Use of MRI is associated with an increase in the initial mastectomy rate in patients planned (prior to MRI) for BCS (OR = 5.18, 95% CI = 2.37 to 11.29) [very low level of certainty]. Approximate initial mastectomy rate: 5.5% versus 1.1%; 4.4% more (95% CI = 3.6% to 11.5% more). Use of MRI is associated with an increase in the final mastectomy rate (OR = 1.87, 95% CI = 1.23 to 2.85) [very low level of certainty]. Approximate final mastectomy rate: 14% versus 8%; 6% more (95% CI = 1.7% to 11.9% more).
- Studies including all patients diagnosed with breast cancer (not restricted to predetermined BCS) showed that use of MRI is associated with an increase in the initial mastectomy rate (OR = 1.29, 95% CI = 1.09 to 1.35) [low level of certainty]. Approximate initial mastectomy rate: 38.0% versus 32.3%, or 5.8% more (95% CI = 1.9% to 9.9% more). The use of MRI is associated with an increase in the final mastectomy rate (OR = 1.19, 95% CI = 1.06 to 1.33). Approximate final mastectomy rate: 41.8% versus 37.6%, 4.2% more (95% CI = 1.4% to 6.9% more). There was no difference in the final mastectomy rate when the trials using registry data were excluded (OR = 0.98, 95% CI = 0.82 to 1.17).

##### Other Supporting Studies (Not Part of the Meta-Analysis)

- A meta-analysis of 22 studies by Brennan et al. found the incremental CBC detection rate over conventional imaging to be 4.1% [27]. This is much higher than the cancer rate of 1.4% in the High Risk Ontario Breast Screening Program [28], in which MRI is routinely used.
- Two studies that characterized mammographically occult ipsilateral lesions (>2 cm away or in different quadrants than the index tumour) found that they were larger than the index lesion in approximately 20% of cases [29,30]. In the absence of MRI, such tumours, unless detected coincidentally during the operation of the index tumour, would be untreated surgically.
- Guidelines by The Canadian Association of Radiologists [16], EUSOBI [13,31], and Blue Shield of California/Blue Cross Blue Shield Association [17,32] have similar recommendations.

#### 3.1.3. Justification for Recommendation 1

- We consider the significant reduction in recurrence, probable improvement in disease-free survival and metachronous CBC, and reduction in reoperations (re-excisions and conversion mastectomies) evidence of benefit that outweighs the potential negative effects overall. This recommendation places a higher value on treating cancer in a single operation and avoiding recurrence than on avoiding the discomfort of an MRI and potential additional biopsies.
- While the absolute benefit is small for most outcomes and not always statistically significant, the trend is toward MRI being beneficial for each outcome, and therefore this consistency strengthens the conclusion that preoperative MRI has a positive impact in general.
- While MRI use is associated with an increase in mastectomy rate, the reasons are likely to be multifactorial, including the need to encompass additional foci of cancer, a lack of BCS/oncoplastic surgery expertise for more complex cases, and patient preferences. In retrospective studies (and some of the RCTs), MRI was used for clinical reasons that may not have been recorded or adjusted for but that could be related to mastectomy use. As mastectomy rates may vary by country, region, hospital, and surgeon, and due to patient factors such as age, relationship status, and race/ethnicity, the additional effect of MRI on mastectomy outcomes is difficult to assess.

### 3.2. Recommendation 2

- Preoperative breast MRI ***is recommended*** in patients diagnosed with invasive lobular carcinoma (ILC) for whom additional information about disease extent could influence treatment. The decision of whether to conduct an MRI should be made in consultation with the patient and must take into account the balance of benefits and risks and patient preferences.

#### 3.2.1. Qualifying Statements for Recommendation 2

- Risks and benefits will vary depending on patient and disease characteristics.
- System issues such as MRI availability may result in treatment delays that may modify the decision.

#### 3.2.2. Key Evidence for Recommendation 2

Evidence for Recommendation 1 would apply, in addition to stronger evidence specifically for ILC:

- Use of MRI is associated with a reduction in the rate of conversion mastectomy in patients with ILC (OR = 0.38, 95% CI = 0.25 to 0.56) [high certainty of evidence]. Approximate conversion mastectomy rate in ILC: 5.9% versus 14.2%; 8.3% fewer (5.7% to 10.3% fewer).
- Use of MRI is associated with a reduction in the rate of positive margins in patients with ILC (OR = 0.63, 95% CI = 0.49 to 0.82) [moderate level of certainty]. Approximate rate of positive margins: 18.9% versus 27.0%; 8.1% fewer (3.7% to 11.7%).
- Use of MRI is associated with a large reduction in the rate of reoperation in patients with ILC (OR = 0.30, 95% CI = 0.13 to 0.72) [moderate level of certainty]. Approximate rate of reoperation: 12.3% versus 31.9%; 19.6% fewer (6.77% to 26.1% fewer).
- Lobbes et al. [33] found MRI increased the detection of synchronous CBC in ILC (OR = 4.07, 95% CI = 1.73 to 3.61, *p* < 0.001) (HR > 1 indicates increased detection with MRI).
- A review of the literature by Mann et al. [34] found synchronous CBC detected only by MRI in 7% of patients (95% CI = 4% to 12%). The recommendation is consistent with guidelines by EUSOBI [13], EUSOMA [12], INESSS [14], and The Royal College of Radiologists (London) [35].

#### 3.2.3. Justification for Recommendation 2

- We consider the significant reduction in positive margins resulting in a large reduction in reoperations (including conversion mastectomy), in addition to the benefits in survival and recurrence for all patients (see Recommendation 1), to be evidence of a benefit that outweighs the potential negative effects overall. This recommendation places a higher value on treating cancer in a single operation and avoiding recurrence than on avoiding the discomfort of an MRI and potential additional biopsies. The benefit of MRI is consistent with the results of studies that reported that, compared to invasive ductal carcinoma, ILC has been found to be more difficult to detect by mammography, more likely multifocal, more often occurs with synchronous CBC, and has more involved margins after initial resection [36,37,38,39,40,41].

### 3.3. Recommendation 3

Preoperative breast MRI **is recommended**, based on the opinion of the Working Group, in the following situations:

(a) To aid in the surgical planning of BCS in patients with suspected or known multicentric or multifocal disease.
(b) To identify additional lesions in patients with dense breasts.
(c) To determine the presence of pectoralis major muscle/chest wall invasion in patients with posteriorly located tumours or when invasion of the pectoralis major muscle or chest wall is suspected.
(d) To aid in surgical planning for skin/nipple-sparing mastectomies or for autologous reconstruction, oncoplastic surgery, and BCS with suspected nipple/areolar involvement.
(e) Patients with familial/hereditary breast cancer who have not had a recent breast MRI as part of screening or diagnosis.

#### 3.3.1. Qualifying Statement for Recommendation 3

Preoperative breast MRI is recommended in the above situations if additional information about disease extent could influence treatment. The decision of whether to conduct an MRI should be made in consultation with the patient and must take into account the balance of benefits and risks and patient preferences.

#### 3.3.2. Key Evidence for Recommendation 3

Comparative studies meeting the evidence review inclusion criteria were not found. These uses are recommended based on the expert opinion of the authors and are consistent with recommendations in other guidelines [12,13,14,17,32,35,42,43]. Some of these situations are implicit in Recommendation 1; however, the authors wanted to draw attention to these uses:

(a) Most studies in the literature review [19] either excluded multicentric and multifocal disease or included these in the list of factors used to adjust results in multivariate analysis, indicating these are known to influence outcomes, but with the result that we did not find a direct comparison of outcomes according to MRI use. The presence of multicentric and multifocal disease increases the complexity of surgical planning and in older guidelines was a contraindication to BCS. When the disease is well-characterized, the possibility of BCS may be increased in some cases and ruled out in others, and the likelihood of an incidental finding during surgery decreases. The consensus of the authors is that the increased sensitivity of MRI justifies its use in suspected/known multicentric or multifocal disease if BCS is desired.
(b) Several studies mentioned in the literature review [19] reported that the sensitivity of mammography decreases as breast density increases, while the sensitivity of MRI is high and independent of breast density. The GEMMA (Gadobutrol-Enhanced MR Mammography) trials studied MRI in patients with newly diagnosed and histologically proven breast cancer. In GEMMA1, MRI sensitivity was 83% (independent of density), while the sensitivity of mammography decreased from 79% to 62% as breast density increased [44]. Corresponding results in the GEMMA2 trial were 91% (independent of density) for MRI and 82% (low density) to 64% (high density) for mammography. The Ottawa study of preoperative MRI found additional lesions changing surgical management in 31% of patients with low density (fat density) and 62% with dense breasts [45]. Screening studies reported similar variations in the sensitivity of mammography based on breast density. The Supplemental MRI Screening for Women with Extremely Dense Breast Tissue (DENSE trial) randomized 40,373 women with extremely dense breast tissue and normal screening mammography to either supplemental MRI or only mammography and found MRI reduced interval cancers by 50% in those offered MRI and 80% in those who agreed to have an MRI [46,47,48]. A systematic review and meta-analysis [49] found that breast density is one of the strongest risk factors for breast cancer.
(c) Tumours near the chest wall may invade the pectoralis major muscle or involve the chest wall, and thus accurate knowledge of tumour extent will influence treatment planning. MRI has been found to have high sensitivity in detecting muscle or chest wall involvement [50,51,52,53].
(d) Standard BCS may lead to fair to poor esthetic and functional results [54], and more complex oncoplastic surgery or mastectomy may be more appropriate if the optimal tumour-to-breast ratio for each quadrant is exceeded. Breast MRI or other advanced imaging (e.g., positron emission tomography/computed tomography) may be a prerequisite for extreme oncoplasty [55]. MRI is frequently used prior to nipple-sparing mastectomy, especially in the case of centrally located tumours [56,57,58,59,60]. MRI may rule out nipple involvement such that 2 cm is no longer considered a minimum tumour-to-nipple distance; 5 mm [61] or 1 cm [62,63,64,65,66,67] may be sufficient.
(e) Hereditary cancer patients have a high risk of synchronous and metachronous CBC. A systematic review reported 10-year CBC rates of 25% to 31% for patients with germline mutations, compared to 4% to 8% for sporadic cases [68].

### 3.4. Technical Factors for MRI Use

MRI is one of the most sensitive imaging techniques for detecting breast tumours, with the potential to be highly specific. Performance depends on the equipment and MRI techniques used and the expertise of those conducting the analysis. The literature review [19] identified several technical documents and standards for MRI use. Guidance on the performance of CE-MRI and biopsies by the Canadian Association of Radiologists [16], ACR [15,69,70,71,72,73,74,75,76,77,78,79], EUSOBI [13,80], and others may be useful; however, these were not critically reviewed or compared in this evidence summary. Several studies used technical standards for MRI set by the American College of Radiology Imaging Network (ACRIN) 6667 trial [81,82,83,84] and EUSOBI, as well as the ACRIN 6698 trial for DWI [85].

Best practice is that additional suspicious lesions detected by preoperative MRI be biopsied or otherwise confirmed if they could alter surgical procedures. Sites performing MRI should have the capacity for an MRI-directed biopsy. This minimizes the need for repeat MRIs and associated costs, delays due to transfer of care (ultimately resulting in a delay in definitive treatment) [86], and the risk of patients not receiving follow-up. Familiarity with the complete process may also result in better expertise in reading and interpreting MRI [87].

## 4. Discussion

In Ontario, there are currently capacity constraints that affect the availability of MRI. Additional MRI use will add system pressure unless capacity issues are resolved and may increase treatment delays beyond what are considered acceptable in some cases. Availability and accessibility vary among regions. Local availability of breast MRI and projected surgical delays due to the addition of preoperative MRI may be major issues in deciding whether MRI is used. Patients indicated that they would like to be aware of these issues and whether they were modifiable in their situation.

Limited availability and high cost are in part due to the long duration of a full MRI scan (30–45 min). Many studies have investigated whether scan time can be reduced without sacrificing sensitivity and specificity or losing other information. As MRI has been found to be beneficial in screening women at high risk of cancer [88,89,90], as well as those at intermediate risk [1,11,91], including patients with dense breasts [46], the majority of evidence comes from screening studies or those enriched in cancerous lesions.

The first major study of abbreviated MRI (AB-MRI) in screening by Kuhl et al. was published in 2014 [92]. Women at mildly to moderately increased risk of breast cancer with negative digital mammography underwent a full diagnostic MRI (8 pulse sequences). For AB-MRI, only the first two sequences (precontrast and first postcontrast acquisition) were read. Acquisition time for AB-MRI sequences was 3 min, compared to 17 min for the full protocol. The additional cancer yield was 18.2/1000. Sensitivity was 100%, and specificity was similar to the full protocol (94.3% vs. 93.9%). Based on this work, many other studies of AB-MRI have been conducted. Specificity was lower in some studies (though generally >80%), and variations in protocol, including additional sequences, have been investigated. Adding a T2-weighted sequence and having at least two post-contrast sequences does not increase the scan time by more than 3 to 4 min and allows improved specificity equivalent to the full protocol. Ultrafast MRI involving a fast post-contrast acquisition capturing the inflow of contrast agent may be used on its own or together with abbreviated MRI; in the latter case, it adds information but does not take additional time [93]. AB-MRI has been reported for over 5400 women in 21 studies published from 2014 to 2018 [94], with an overall sensitivity of 94% and a specificity of 90%. A later review identified 41 studies until 2020 involving 15,680 MRI examinations [95]. There is not a common definition of AB-MRI, and it sometimes refers to just the precontrast and postcontrast sequences, sequences less than 7 to 10 min, or any protocol that is significantly shorter than the standard (full) MRI protocol.

The ACR accreditation requirements for breast MRI include a precontrast sequence (T2 weighted/bright fluid series, multi-phase T1-weighted series, and pre-contrast T1; these may be separate or combined), and early postcontrast and delayed postcontrast T1-weighted sequences [96]. Massachusetts General Hospital (Boston, Massachusetts) has used a rapid abridged multiphase (RAMP) breast MRI protocol since 2016 that meets ACR requirements and has a scan time of 10 min [97].

In Ontario, use of the full diagnostic protocol is common and requires 30 to 45 min. Some cancer centres, including those in Ottawa and London, use a shortened protocol that requires a scan time of 12 min and meets Canadian Association of Radiologists [16] and Ontario Breast Screening Program guidelines. As shorter protocols become more standardized and implemented, there is potential for cost reduction and increased patient scans. It is acknowledged that personnel, time for setup, and interpretation of results may be limiting factors until the entire workflow is rebalanced.

### 4.1. Limitations

This literature review referred to in this guideline included primarily retrospective studies that may have additional confounding factors for which adjustments were not made. While the benefits of MRI use in these studies are generally consistent, the magnitude of the benefit is less certain due to differences in patient populations, study designs, and methods of adjustment for confounders. Comparative studies on the use of MRI versus no MRI that met our inclusion criteria were not found for many of the subgroups of interest, including the use of systemic therapy or radiotherapy. Cost analysis was outside the scope of this work.

### 4.2. Review and Update

The currency of each document is ensured through periodic review and evaluation of the scientific literature and, where appropriate, the addition of newer literature to the original evidence base. This is described in the *PEBC Document Assessment and Review Protocol*. For the full 1–25 guideline, systematic review, and subsequent updates, please visit the OH (CCO) website at https://www.cancercareontario.ca/en/guidelines-advice/types-of-cancer/breast (accessed on 1 May 2023).

## Figures and Tables

**Table 1:** Potential benefits and harms of preoperative MRI.

Factor	Potential Benefits	Potential Harms
**High** **Sensitivity**	MRI is not impacted by breast density, which limits the sensitivity of mammography. Higher cancer detection rates with MRI than mammography, with greater ability to detect occult cancer in the ipsilateral breast with multifocal and multicentric disease. More accurate staging of the contralateral breast reduces the rate of breast cancer detected in follow-up. Allows the detection of all cancerous lesions at the start so they can be treated at one time instead of having pre-existing cancers only detected on short-term follow-up; this can have cost benefits for patients and the health care system, reduce anxiety, and improve the quality of life of patients. Confirmation of limited disease may allow for more conservative treatment such as partial breast irradiation (including in patients with previous radiotherapy) or the omission of systemic therapy. May allow a longer interval between initial treatment and follow-up imaging. Additional information from MRI reduces the frequency of reoperations to achieve clear margins and reduces the rate of unplanned (salvage) mastectomy subsequent to the initial BCS. This can have cost benefits for patients and the health care system, reduce surgical complications, reduce anxiety, and improve the quality of life of patients. May confirm or rule out the feasibility of nipple-sparing mastectomy. In the setting of Paget disease with negative conventional imaging studies, MRI can identify underlying breast malignancy, facilitating proper treatment planning.	Higher breast biopsy rates, including some lesions that will be negative for cancer (i.e., false-positive by MRI). Higher mastectomy rates with MRI when disease extent is greater than shown on conventional imaging. Repeat (short-interval follow-up) MRIs may be required for BI-RADS 3 lesions if an MRI-guided biopsy was not conducted or with benign breast biopsies. More aggressive surgery or other treatment due to knowledge of additional lesions may not change survival outcomes. MRI is not necessarily more accurate in estimating tumour size than other imaging; the optimal modality may vary with tumour characteristics.
**Specificity**	Specificity is generally greater than 70%, and up to 97% has been reported [1]. MRI specificity depends on study populations, technical methods, and criteria for interpretation.	Specificity may be lower than mammography in some MRI centres or for some applications. MRI-detected lesions require biopsy for tissue confirmation and may include false-positive lesions.
**Patient** **Factors**	May reduce the mastectomy rate in patients initially opting for mastectomy due to fear of more extensive disease and not due to clinical factors. Reduction in anxiety for some patients as they are more confident regarding the appropriateness of treatment planned or received.	Some patients are not suitable for MRI (anxiety, claustrophobia, MRI does not accommodate body habitus or other patient concerns) or do not want to undergo this procedure. Increased anxiety for some patients regarding MRI procedures or biopsies, or while waiting for these to occur or results to be reported.
**Adverse** **Effects**		Gadolinium contrast agents may cause allergic reactions (≈0.1% of patients). Gadolinium retention, especially after multiple MRIs, has been reported in the brain; long-term effects are uncertain but have not been reported to date. Accumulation depends on the type of contrast agent and cumulative exposure. Nephrogenic systemic fibrosis may occur in patients with acute kidney injury or severe chronic kidney disease; the risk varies with the type and volume of gadolinium contrast agent used.
**Delay in Treatment**		Breast MRI use may potentially lead to delays in treatment due to both MRI scheduling and the characterization of any identified lesions (biopsies and histopathology analysis/reporting). May increase anxiety for patients while waiting for treatment.
**Equity**	Universal access to preoperative MRI would result in more health care equity, provided equivalent facilities and staffing are available.	Breast MRI, including expertise for interpretation, is not available in all centres, and some patients may need to travel long distances.
**Cost**	Better lesion characterization may reduce operative costs by reducing rates of reoperations (direct surgical costs for multiple operations, treating surgical complications, patient time), costs to treat metachronous contralateral breast cancer, and longer-term costs due to decreased recurrence.	The addition of an MRI and subsequent biopsy of lesions will add to the initial diagnostic cost.

**Abbreviations:** BCS, breast conserving surgery; BI-RADS, Breast Imaging Reporting and Data System; MRI, magnetic resonance imaging.
